# HIV-Associated Hodgkin's Lymphoma: Prognosis and Therapy in the Era of cART

**DOI:** 10.1155/2012/507257

**Published:** 2012-01-05

**Authors:** Caron A. Jacobson, Jeremy S. Abramson

**Affiliations:** ^1^. Dana-Farber Cancer Institute, Boston, MA 02215, USA; ^2^Center for Lymphoma, Massachusetts General Hospital Cancer Center, Boston, MA 02114, USA

## Abstract

Patients with human immunodeficiency virus/acquired immune deficiency syndrome (HIV/AIDS) are at increased risk for developing Hodgkin's lymphoma (HL), a risk that has not decreased despite the success of combination antiretroviral therapy (cART) in the modern era. HIV-associated HL (HIV-HL) differs from HL in non-HIV-infected patients in that it is nearly always associated with Epstein-Barr virus (EBV) and more often presents with high-risk features of advanced disease, systemic “B” symptoms, and extranodal involvement. Before the introduction of cART, patients with HIV-HL had lower response rates and worse outcomes than non-HIV-infected HL patients treated with conventional chemotherapy. The introduction of cART, however, has allowed for the delivery of full-dose and dose-intensive chemotherapy regimens with improved outcomes that approach those seen in non-HIV infected patients. Despite these significant advances, HIV-HL patients remain at increased risk for treatment-related toxicities and drug-drug interactions which require careful attention and supportive care to insure the safe administration of therapy. This paper will address the modern diagnosis, risk stratification, and therapy of HIV-associated HL.

## 1. Introduction

Since the introduction of combination antiretroviral therapy (cART) in 1996, patients with human immunodeficiency virus (HIV) infection are living longer, with improved immune function and a reduced risk of developing acquired immune deficiency syndrome (AIDS) [1, 2]. In concert with improved viral control, there has been a substantial change in the landscape of malignancies occurring in the setting of HIV. AIDS-defining cancers such as Kaposi's sarcoma (KS) and non-Hodgkin's lymphomas (NHL) have declined significantly, though the change in NHL incidence has not applied evenly across disease subtypes. Diffuse large B-cell lymphoma, primary CNS lymphoma, plasmablastic lymphoma, and primary effusion lymphoma have all declined, while Burkitt lymphoma has remained stable. Over the same time period, non-AIDS-defining malignancies, including numerous solid tumors and Hodgkin's lymphoma (HL), have remained stable or have increased in incidence [3, 4]. The growth of an aging population with HIV has contributed to this rise, but the risk of many of these cancers remains significantly increased above that observed in the general population, suggesting an effect of ongoing virus-mediated immune suppression and stimulation on cancer risk despite the salutary effects of antiretroviral therapy [3–8].

There will be approximately 8800 new cases of HL diagnosed this year in the United States and 1300 deaths [9]. HL outside of HIV is a disease characterized by a bimodal age distribution with an initial peak at age 20–30 and a second peak at age 50–65 [10], while the median age of HL presentation in HIV is in the 30s [11]. The incidence of this disease in HIV-positive patients is 5–10 times higher than in the general population, and may be increasing since the introduction of cART [3–8, 12, 13]. Histologically, the malignant Hodgkin Reed-Sternberg (HRS) cells comprise less than 1% of the tumor cellularity, with the majority made up of surrounding polyclonal lymphocytes, eosinophils, neutrophils, macrophages, plasma cells, fibroblasts, and collagen. The HRS cell interacts with its microenvironment via cell-cell contact and elaboration of growth factors and cytokines, which results in a surrounding cellular milieu that protects it from host immune attack. The surrounding environmental cells likewise support the HRS cells via cell-cell signaling and cytokine production which provides the necessary signals that promote proliferation and survival of the HRS cell itself. Severe immunosuppression, as in advanced HIV/AIDS, may disrupt this productive relationship with the host microenvironment, resulting in a decreased incidence of HL in the setting of profound immunosuppression. This may explain why the incidence of HL in HIV peaks at a modestly decreased CD4 count (150–199 cells/*μ*L), and disease risk is associated with cART, but it is rarely seen at severely depressed CD4 counts [4, 14, 15]. While the introduction of cART has not resulted in a decreased incidence of this disease, it has resulted in significantly improved outcomes following treatment, resulting largely from decreased treatment-related morbidity and mortality, the ability to treat with full-dose chemotherapy regimens, and an increasing incidence of lower-risk disease. As such, patients with HIV-HL now enjoy similar response rates and progression-free and overall survivals to their stage- and risk-matched non-HIV infected counterparts.

## 2. Epidemiology and Pathology

The epidemiologic and pathologic pattern of HIV-associated HL is distinct from that observed in HIV-negative patients. There is an increased risk of developing HL in HIV-infected patients compared to the general population, and this risk remains increased since the advent of cART [3–8, 12, 13]. In a prospective cohort of 11,112 HIV-positive patients, the incidence of HL was nearly 14 times higher than that of the general population, with variation based on the era of diagnosis; standardized incidence was 4.5 times higher than the general population in the pre-cART era (1983–1985) compared to 32 times higher in the cART era (2002–2007) [4]. These observations were replicated in the Swiss HIV Cohort Study of 9429 HIV-infected patients, where HL standardized incidence was 9 times increased in the pre-cART era, compared with 21 times increased in the early cART era and 28 times increased in the late cART era [7]. This is consistent with multivariate analysis revealing that cART and specifically nonnucleoside reverse transcriptase inhibitors are associated with an increased risk of developing HIV-HL [4]. In addition, the pattern of histologic subtypes of HL seen in HIV-infected patients differs from the general population, with a greater proportion of mixed cellularity (MC) and lymphocyte depletion (LD) observed in the former [16, 17]. MC and LD subtypes of classical HL are correlated with more advanced immune compromise, while nodular sclerosis (NS) histology increases with higher CD4 counts and use of cART [15].

Viral oncogenesis appears to play a greater role in HIV-HL than HL in the general population. Epstein-Barr virus (EBV) can be detected in approximately one-third of cases of non-HIV-associated HL, compared to nearly all cases of HIV-HL [11, 15]. HRS cells in HIV-HL express the EBV-transforming protein latent membrane protein 1 (LMP-1), and the EBV genomes from multiple disease sites in the same HIV-HL patient are episomal and clonal, suggesting that EBV is directly involved in lymphomagenesis [18–21].

## 3. Clinical Presentation and Prognostic Factors

**Figure 1:**
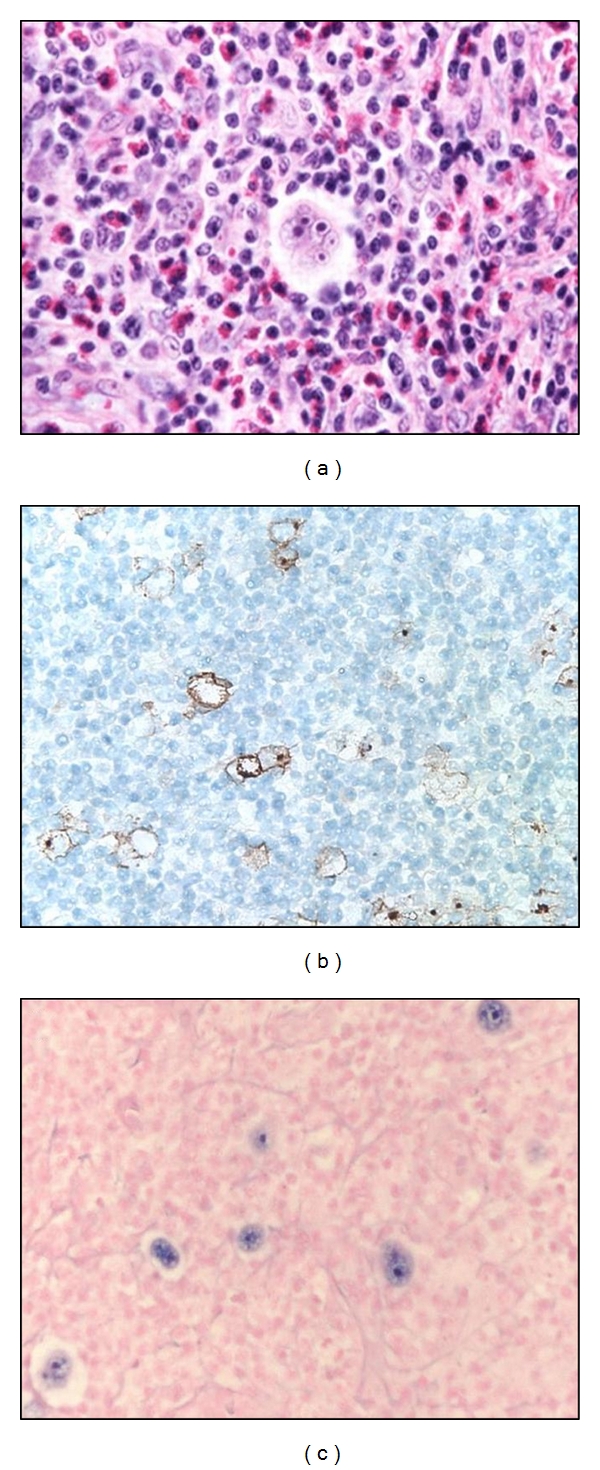
Representative images of mixed cellularity HIV-associated classical Hodgkin's lymphoma. (a) High-power H and E image shows a prominent Hodgkin Reed Sternberg (HRS) cell surrounded by a mixed population of lymphocytes, eosinophils, granulocytes, and histiocytes. (b) Low-power view of CD30 immunohistochemistry highlights the rare large HRS cells, as does in-situ hybridization for EBER (c), reflecting the Epstein-Barr virus (EBV) infection of HRS cells.

HIV-HL in the modern era presents at a median age in the mid 30s after a median time from HIV diagnosis of approximately 7 1/2 years. Approximately one quarter of patients will have a prior AIDS diagnosis at the time their cancer is diagnosed, and the majority of patients will be diagnosed while receiving cART. The median CD4 count at HL diagnosis is approximately 240 cells/*μ*L. The most common histology is MC in approximately half of patients, followed by NS in one quarter and LD in approximately 10%. Representative pathologic images are shown in Figure 1. The majority of patients present with advanced-stage disease (Ann Arbor stage III-IV), though the incidence of early-stage disease appears to be increasing in the cART era. The majority of patients still present with systemic “B” symptoms, and extranodal involvement remains common [11, 17, 22–24].

Prior to the availability of cART, prognosis of HIV-HL was poor with very few patients cured of their disease. A number of adverse prognostic factors were identified, including MC subtype, extranodal involvement, presence of systemic “B” symptoms, and a high International Prognostic Score (IPS) [11, 25]. The development of cART has significantly changed the natural history and risk stratification of HIV-HL. The Spanish GESIDA group compared 83 patients with HIV-HL treated with cART to 21 patients with HIV-HL not treated with cART and found similarly high-risk clinical characteristics in the two groups at baseline but with significantly better outcome in patients receiving cART, including a higher complete remission (CR) rate (91% versus 70%) and longer median OS longer (not reached versus 39 months) [26]. A CD4 count >100/*μ*L and use of cART were independently associated with a favorable outcome. Other studies have documented the importance of cART on prognosis with improved responses to chemotherapy and survival in the cART era [27, 28]. Importantly, it appears that responding to cART is significant as patients who fail to respond to cART have similarly poor outcomes as patients in the pre-cART era [28].

## 4. Initial Staging and Evaluation

Regardless of an underlying HIV diagnosis, all patients with newly diagnosed HL require a careful medical history including questions regarding systemic “B” symptoms, and a physical exam with attention paid to both nodal and extranodal sites. Laboratory evaluation should include a complete blood count with differential, erythrocyte sedimentation rate, and chemistry tests including a complete metabolic panel with liver function tests and albumin. Baseline HIV parameters including CD4 count and viral load should be tested, along with hepatitis B and C serologies given risk of coinfection. Patients with any HIV-associated malignancy may present with concurrent opportunistic infections or other HIV-associated malignancies, so any findings not readily ascribed to the HL should be evaluated further as clinically indicated.

Initial radiographic staging is increasingly with Positron Emission Tomography/Computed Tomography (PET/CT) scans, which are associated with a higher sensitivity, specificity, and positive and negative predictive value than traditional CT scans in the initial staging of HL [29]. They may also be useful in evaluating for bone marrow involvement, which is often patchy in HL, though false positives do occur [30]. The value of PET/CT scans for staging specifically in patients with HIV-associated HL has not been studied and is less clear than that in the non-HIV-infected patients due to a higher rate of false positives owing to the competing infectious, inflammatory, and/or malignant processes that may produce PET-avid lesions in immunosuppressed patients. PET certainly adds to the sensitivity in detecting extranodal sites of disease, which are present in the majority of patients with HIV-HL and may be missed on conventional CT scanning. Bone marrow aspiration and biopsy should generally be performed at diagnosis and repeated following therapy for confirmation of CR only if positive at presentation. Patients with HIV/AIDS are at increased risk for cardiovascular and pulmonary disease of multiple etiologies, and these organs may also be injured by chemotherapy; as a result, all patients should have a pre-treatment assessment of cardiac function (echocardiogram or multigated acquisition scan) and pulmonary function tests, given the risks associated with doxorubicin and bleomycin, respectively.

## 5. Initial Treatment

The most commonly used initial systemic therapy for HL worldwide is the combination of doxorubicin, bleomycin, vinblastine, and dacarbazine (ABVD). When ABVD was given to patients with HIV-HL in the pre-cART era, the objective response rate was a disappointing 62% with a median OS of only 1.5 years [31]. Even though the majority of these patients had high-risk features at diagnosis including stage IV disease, bone marrow involvement, and “B” symptoms, these results are markedly inferior to outcomes expected in high-risk patients with HL not associated with HIV. Despite all patients receiving granulocyte-colony-stimulating factor (G-CSF), over half of these patients experienced significant neutropenia requiring treatment delays, and the incidence of opportunistic infections, despite prophylactic antimicrobials, was 29%, with the same percentage of patients experiencing fatal infections during the study period. A slightly higher response rate but shorter OS was observed when epirubicin, bleomycin, and vinblastine were given to HIV-HL patients before the introduction of cART, but most of the responders were patients with a better performance status and without a history of opportunistic infections [32]. Decreased response rates, due to dose reductions and delays, higher risk disease, and increased treatment-related morbidity and mortality all contribute to the inferior outcomes during this era.

The introduction of cART and its associated improved control of HIV infection, immune status, and performance status have allowed for administration of full-dose-intensive regimens with improved outcomes (Table 1). In the early cART era, the treatment of 35 patients with advanced high-risk HIV-HL with EBV plus prednisone (EBVP) and either zidovudine or dideoxyinosine resulted in an overall response rate of 91%, but still with a disappointing median overall survival of 16 months and a 3-year OS and DFS of 32% and 53%, respectively [33], highlighting an ongoing high rate of nonrelapse mortality. The delivery of chemotherapy with cART proved feasible, however, and demonstrated an increased ability to cure HL in a subset of these very high-risk patients.

As cART has evolved, treatment results in HIV-HL have further improved. A prospective phase 2 study of the Stanford V regimen with cART in 59 patients with HIV-HL found that less than one-third of patients required a dose reduction or delay [35]. These patients had better risk disease than those reported previously, with a greater proportion of patients with a good performance status and early-stage disease, and fewer patients with extranodal involvement and “B” symptoms. The median pre-treatment CD4 count was 238 cells/*μ*L. Eighty-one percent of patients achieved a CR, with a 5-year OS and DFS of 59% and 68%, respectively. An IPS <2 was associated with improved freedom from progression. The more intensive regimen of bleomycin, etoposide, doxorubicin, cyclophosphamide, vincristine, procarbazine, and prednisone (BEACOPP) similarly resulted in a high rate of CR in all 12 HIV-HL patients in a small report [36]. Two-thirds of these patients had a good performance status and the median CD4 count was 205/*μ*L, but there was a greater proportion of advanced-stage disease, “B” symptoms, and extranodal involvement than that in the European Intergroup study of Stanford V. After 4 years, there had only been one relapse, but the incidence of severe neutropenia was 75%, and two patients died from opportunistic infections.

ABVD was reexamined in conjunction with cART in a cohort of 62 high-risk HIV-HL patients, 87% of whom achieved a CR, with an encouraging 5-year OS and event-free survival (EFS) of 76% and 71%, respectively [34]. An immunologic response to cART was associated with improved outcome. Finally, GICAT explored the use of epirubicin, bleomycin, vinorelbine, cyclophosphamide, and prednisone (VEBEP) in 71 patients with HIV-HL, many of whom had advanced-stage and high-risk disease by IPS [37]. The CR rate was 67%, with 69% of patients alive and 86% of patients disease-free at 2 years.

As noted earlier, the introduction of cART has resulted in an increased number of HIV-HL patients presenting with earlier-stage disease. The optimum therapy for early-stage HL is controversial and an area of active investigation. Combined modality therapy with chemotherapy and radiation has been the standard of care, but late complications of radiotherapy including secondary malignancies and heart and lung disease have prompted consideration of chemotherapy alone in selected patients. Randomized trials in nonbulky limited-stage disease have identified no survival benefit favoring inclusion of radiotherapy, and retrospective analyses of chemotherapy alone show encouraging results, so avoiding radiotherapy is an option for limited stage patients without presenting bulk, with the caveat that HIV-HL patients have not been included in these studies [45–47]. Although this has not been studied in patients with early-stage HIV-HL, it is reasonable to approach the treatment of early stage HIV-HL similarly to that of HL patients without concomitant HIV infection, with appropriate attention to supportive care.

These data demonstrate that HIV-HL patients treated with concurrent cART in the modern era achieve similarly encouraging results as those seen in the general population when disease risk factors are matched. The improved outcomes in patients with HIV-HL demonstrate the importance of improved immunologic status and performance status, and the ability to treat on schedule and at full dose intensity in the modern era.

## 6. Relapsed and Refractory Disease

Despite improved outcomes with initial therapy, a number of HIV-HL patients still relapse, for whom prognosis is poor. High-dose chemotherapy with autologous stem cell transplantation (HDC-ASCT) remains the standard of care in HIV-negative patients with relapsed HL based on improved PFS and EFS compared to traditional salvage chemotherapy [48, 49]. In the cART era, HDC-ASCT has been shown to be a feasible and successful strategy in relapsed or refractory HIV-HL as well, but with significant potential toxicity [38–44, 50] (Table 2). A prospective study of HDC-ASCT as salvage therapy for AIDS-related lymphomas included 50 patients, 24 of whom actually received the planned HDC-ASCT [50]. The median OS for the entire cohort was only 7 months, but the median OS for patients undergoing transplantation had not been reached at 44 months, demonstrating that a favorable outcome can be achieved in selected cases of relapsed HIV-HL. These findings are supported by a retrospective analysis of HDC-ASCT in relapsed HIV-associated lymphoma patients (one-third of whom had HL), where PFS and OS were similar to an HIV-negative cohort matched for disease risk factors [44]. The incidence of grade 3 and 4 toxicities following HDC-ASCT for HIV-HL is approximately 30–40%, including upper and lower gastrointestinal toxicity, hepatotoxicity, and neutropenic infections [38–44, 50]. In addition, the rate of viral reactivation and infections with cytomegalovirus, herpes zoster virus, and/or varicella zoster virus is 10–25%, and 5–7% for fungal infections; these are similar to that observed following HDC-ASCT in non-HIV patients [38–44, 50, 51]. The rate of transplant-related mortality is also comparable to that seen following autologous transplantation in patients not infected with HIV and ranges from 0 to 5% across available studies [38–44, 50]. For appropriately selected patients, HDC-ASCT is feasible and effective salvage therapy for relapsed or refractory HIV-HL, but this should only be performed at transplant centers experienced in the administration of high-dose chemotherapy to HIV-infected individuals.

The data exploring the use of allogeneic stem cell transplant are retrospective or based on case reports. The Center for International Blood and Marrow Transplantation Research reported the experience of 27 HIV-associated lymphoma patients treated with allogeneic transplantation from 1986 to 2003 [52]. Two-year OS was only 22% in this group, although survival was improved in the post- compared with pre-cART era. Given the limited experience and high-risks of this approach, allogeneic stem cell transplantation should be considered experimental and optimally performed in the setting of a clinical trial.

For patients who are not candidates for, or who have relapsed after, HDC-ASCT, traditional chemotherapy agents remain available as monotherapy or in combination. While these therapies may induce remissions, this is without significant opportunity for cure and is associated with ongoing chemotherapy toxicities and immune suppression. Novel agents are now becoming available for relapsed/refractory HL, though they have not been studied to date in the HIV-HL population. Brentuximab vedotin (SGN-35) is a monoclonal antibody against CD30 that is bound to the microtubule toxin monomethyl auristatin E (MMAE) and has recently been FDA approved for the treatment of Hodgkin lymphoma that has relapsed after HDC-ASCT, or in patients ineligible for ASCT. In non-HIV-infected HL patients, all of whom had failed prior HDC-ASCT, the overall response rate was a remarkable 75% with 34% of patients achieving a CR, many of which appeared durable [53]. This novel targeted therapy emerges as an appealing chemotherapy-sparing treatment option for relapsed HIV-HL patients who have relapsed after, or are not candidates for, high-dose chemotherapy. Additional novel agents are currently under investigation and appear promising in HL, including mTOR inhibitors and histone deacetylase inhibitors, among others.

## 7. Restaging and Follow-Up

Following completion of therapy, restaging with PET/CT scans in non-HIV-infected HL patients is better at differentiating between viable and necrotic/fibrotic tumor than traditional CT scans and has a higher positive and negative predictive value [54]. In addition, an interim PET/CT response after 2-3 cycles of chemotherapy carries significant prognostic value in this disease, although patients who convert to PET negative at the end of therapy have been shown to do similarly well to those who are PET negative mid-therapy [55–58]. There is no evidence to date, however, that interim PET/CT results can be used to alter treatment plans, and this is being evaluated in a number of ongoing clinical trials. PET/CT for lymphoma restaging should also be interpreted with some caution in the setting of HIV, as they appear to be less specific for persistent disease than in non-HIV-infected patients [59]. Positive PET scans should, therefore, prompt tissue sampling to confirm persistent or recurrent disease prior to altering therapy for presumed treatment failure. At present, surveillance PET/CT scans should not be performed in routine follow-up after patients achieve a CR, where CT scans alone remain sufficient.

## 8. Toxicity and Supportive Care

While the use of cART in combination with full-dose chemotherapy has resulted in improved clinical outcomes, HIV-positive HL patients remain at significantly increased risk for treatment-related complications, including infections and drug toxicity. Drug interactions between antiretroviral medications and chemotherapy may lead to increased levels and toxicity of some agents, while others may become subtherapeutic [60]. Numerous antiretroviral drugs, particularly the nonnucleoside reverse transcriptase inhibitors, serve as inducers of the cytochrome P450 (CYP450) system, while others, especially the protease inhibitors, inhibit CYP450. Manipulation of the CYP450 system affects the metabolism of both antiretroviral drugs and chemotherapy agents. Multiple chemotherapies, including doxorubicin, dacarbazine, vinblastine, and etoposide, are metabolized by the CYP450 system, and as such their levels may be increased or decreased in the setting of CYP450 inhibition or induction, respectively. This may lead to enhanced myelosuppression, as well as increased risk of neuropathy related to increased vinblastine levels. Stavudine and didanosine are likewise associated with neurotoxicity and should be avoided when treating with a vinca alkaloid or other neurotoxic chemotherapies (taxanes and platinums). There is evidence that use of chemotherapy and zidovudine, with its affect on myelopoiesis, and/or protease inhibitors, with their potent inhibition of CYP3A, results in greater myelotoxicity and prolonged neutropenia; avoidance of these drugs should be considered, if possible [61]. The routine, prophylactic use of G-CSF and *Pneumocystis jiroveci* prophylaxis (regardless of the CD4 count prior to treatment) is recommended for all patients to minimize the extent of myelosuppression and the risk of infection in these immunosuppressed patients. Additionally, providers should pay careful attention to pulmonary symptoms during treatment, as the effect of HIV disease and associated opportunistic infections of the lungs may potentiate the pulmonary toxicity of bleomycin. Caution should also be taken in dosing of hepatically cleared chemotherapy agents such as vinca alkaloids and doxorubicin based on bilirubin levels. Certain protease inhibitors, most notably atazanavir and indinavir, cause an indirect hyperbilirubinemia due to inhibition of UGT1A1 in the liver, but this does not affect drug clearance. An indirect hyperbilirubinemia with a normal direct bilirubin and absence of other findings of hepatotoxicity should, therefore, not prompt dose reductions of chemotherapy drugs. These risks notwithstanding cART can clearly be administered safely in combination with chemotherapy, even with dose-intensive regimens, with acceptable toxicity profiles [32–37, 62, 63]. Careful attention, however, must be paid to potential drug-drug interactions and toxicities. Given the increased risk of toxicities due to drug-drug interactions but the clear benefit of administering combination chemotherapy concurrently with cART, these patients should ideally be cared for by oncologists experienced in the care of HIV-associated malignancies, and their care requires close collaboration of a multidisciplinary care team including oncologists and infectious disease specialists.

## 9. Conclusions

Although the incidence of HIV-HL has not declined in the decades since the introduction of cART, the prognosis has significantly improved and is now analogous to their risk- and stage-matched HIV-negative counterparts when treated with full-dose chemotherapy and concurrent cART. The lack of randomized trials in this disease makes it difficult to identify an optimum regimen for the upfront treatment of these patients, but ABVD appears to be efficacious and well tolerated, even in high-risk patients. Further study is needed to compare treatment regimens and to validate the use of PET/CT scans in the staging, interim restaging, and post-treatment evaluation of HIV-HL. In addition, the promising experience of novel therapies like brentuximab vedotin and others will ideally be tested specifically in HIV-infected patients. Finally, evaluation of long-term and late-treatment-related toxicity is needed in patients with HIV-HL due to the increasing success of our therapies and the encouragingly long survivals of these patients in the modern era.

## Figures and Tables

**Table 1:** Prospective studies of combination chemotherapy for HIV-HL in the cART era.

Regimen	*N*	Initial CD4 Count/*μ*L,	Advanced stage	Extranodal disease	“B” symptoms	Response rate	Overall survival
EBV [32]	17	184	88%	77%	82%	82%	48% (36 m)
EBVP [33]	35	219	83%	84%	89%	91%	32% (36 m)
ABVD [34]	62	129	100%	N/R	89%	87%	76% (60 m)
Stanford V [35]	59	238	71%	47%	75%	89%	51% (36 m)
BEACOPP [36]	12	205	92%	42%	83%	100%	75% (36 m)
VEBEP [37]	71	248	70%	NR	NR	79%	69% (48 m)

EBV: epirubicin, bleomycin, vinblastine; EBVP: epirubicin, bleomycin, vinblastine, prednisone; ABVD: doxorubicin, bleomycin, vinblastine, dacarbazine; BEACOPP: bleomycin, etoposide, doxorubicin, cyclophosphamide, vincristine, procarbazine, prednisone; VEBEP: vinorelbine, epirubicin, bleomycin, cyclophosphamide, prednisone; NR: not reported.

**Table 2:** Studies of high-dose chemotherapy with autologous stem cell transplantation in relapsed HIV-associated lymphomas.

Study	*N*	%HL	cART	Complete response	Disease-free survival	Overall survival	Treatment-related mortality
Gabarre et al. [38]	14	43%	Yes	71%	29% (26 m)	36% (32 m)	0%
Krishnan et al. [39]	20	10%	Yes	90%	85% (32 m)	85% (32 m)	5%
Serrano et al. [40]	14	21%	Yes	73%	65% (30 m)	65% (30 m)	0%
Spitzer et al. [41]	20	25%	Yes	53%	49% (6 m)	74% (6 m)	5%
Balsalobre et al. [42]	68	26%	Yes	NR	56% (32 m)	61% (32 m)	4%
Re et al. [43]	50 (27*)	38%	Yes	48% (89%*)	49% (44 m) (74% (44 m)*)	50% (44 m) (75% (44 m)*)	0%
Díez-Martín et al. [44]	53	34%	Yes	NR	61% (30 m)	62% (30 m)	NR

NR: not reported.

*Indicates results for only patients who received high-dose chemotherapy followed by autologous stem cell transplantation.
